# GABA levels decline with age: A longitudinal study

**DOI:** 10.1162/imag_a_00224

**Published:** 2024-07-15

**Authors:** Mark D. Zuppichini, Abbey M. Hamlin, Quan Zhou, Esther Kim, Shreya Rajagopal, Adriene M. Beltz, Thad A. Polk

**Affiliations:** Department of Psychology, University of Michigan, Ann Arbor, MI, United States; Department of Psychology, The University of Texas at Austin, Austin, TX, United States

**Keywords:** GABA, aging, longitudinal design, MRS

## Abstract

One factor that might contribute to functional deterioration in healthy older adults is a decline in the brain’s major inhibitory neurotransmitter, gamma-aminobutyric acid (GABA). Previous studies have reported mixed results regarding whether GABA declines in healthy aging. These previous studies were cross-sectional and therefore cannot provide insight into GABA changes over time within aging individuals. Furthermore, aging is associated with gray and white matter atrophy that may confound age-related differences in GABA. In the present study, we utilized a repeated-measures, longitudinal design and MR spectroscopy to measure GABA levels in bilateral auditory, sensorimotor, and ventrovisual voxels of interest (VOI) in 30 healthy older adults at two time points a few years apart. Furthermore, we applied two of the most common tissue correction strategies to control for the effects of tissue composition on GABA estimates. Results from mixed-effects models showed that longitudinal change in age is a significant predictor of tissue-corrected longitudinal change in GABA levels: as age increases, GABA declines. In contrast, there was no cross-sectional effect of age on GABA in our sample (e.g., the oldest old did not have lower GABA levels than the youngest old). In conclusion, results from this study provide support for within-person, age-related declines in GABA over time, even after controlling for tissue composition.

## 1 Introduction

Advanced age is associated with declines in sensory, motor (Schneider & Pichora-Fuller, 2000;Stelmach & Hömberg, 2012), and cognitive function (Harada et al., 2013;D. C. Park et al., 2002;Salthouse, 2012) that negatively impacts vocational ability and quality of life (Seidler et al., 2010). Age-related declines in sensorimotor and cognitive function have been associated with alterations in gray matter, white matter, functional organization, and neurochemistry (Cassady et al., 2019;Goh et al., 2010;Raz & Rodrigue, 2006;Seidler et al., 2010). Such declines vary substantially across individuals and might serve as early indicators of pathological aging (Wilson et al., 2002). Therefore, a better understanding of neural changes underlying age-related functional decline has implications for prolonging functional independence late in life and for the earlier detection, prevention, and treatment of age-related pathology.

The brain’s major inhibitory neurotransmitter, γ-aminobutyric acid (GABA), is one factor that previous research suggests might contribute to age-related functional decline. The GABA signaling system is critical for the responsiveness, excitability, and synchronization of cortical neuronal signaling throughout the mammalian central nervous system and has been shown to play a central role in the regulation of motor function, neural development, cognition, learning, and memory (Govindpani et al., 2017). Disruption of the GABA signaling system has been posited to be an underlying factor in the pathogenesis of several neurological disorders, such as epilepsy (Cossart et al., 2005), major depressive disorder (Luscher et al., 2011), anxiety (Möhler, 2012), autism (Pizzarelli & Cherubini, 2011), schizophrenia (Guidotti et al., 2005), and Alzheimer’s disease (Govindpani et al., 2017;Rissman et al., 2007).

The GABA signaling system consists of several components that are liable to aging effects. GABA is synthesized by the ɑ-decarboxylation of L-glutamate by the enzyme glutamic acid decarboxylase (GAD), of which two isoforms exists: GAD65, which has been shown to be localized to the synaptic boutons and plays a role in the synthesis of GABA released via vesicular mechanisms; and GAD67, which has been localized to cytosol and plays a role in the synthesis of cytoplasmic GABA (Belhage et al., 1993;Soghomonian & Martin, 1998). GABA is metabolized and regulated by astrocytes via the glutamate/GABA-glutamine cycle (Bak et al., 2006). Following neurotransmission, GABA is taken up by tightly enveloped astroglial processes and catabolized to succinate by GABA transaminase and succinate semialdehyde dehydrogenase. Succinate is converted to glutamine via the tricarboxylic acid cycle by glutamine synthase and transported into neurons where it is converted to glutamate and readily available for GABA synthesis by GAD (Patel et al., 1970;Peng et al., 1993).

Given its role in the synchronization of neuronal activity, the timing of receptor activation is paramount for GABA-signaling. Four GABA/NA/Cl transporters are the primary mediators of GABA transport: GABA transporter 1 (GAT1), GAT2, GAT3, and betaine-GABA transporter (BGT1). Of these, GAT1 is the primary presynaptic transporter in the human CNS, whereas GAT2 is primarily found outside the CNS, GAT3 is primarily found in astrocytes rather than neurons, and BGT1 has a higher affinity for betaine and thus primarily transports betaine (Minelli et al., 1995). Vesicular GABA transporter (vGAT) is responsible for intracellular transport of GABA into vesicles. Receptors of GABAergic neurons fall within two classes: ionotropic GABA_A/C_receptors and metabotropic GABA_B_. GABA_A/C_ionotropic receptors are largely responsible for the inhibition and modulation of fast synaptic transmission, whereas GABA_B_metabotropic receptors act as G-protein coupled receptors.

Evidence of an aging effect has been observed on various aspects of the GABA signaling system. For example, age-related decreases in levels of GAD, GABA neurotransmitter levels, GABA neurons, and GABA receptors have been observed in inferior colliculi (Caspary et al., 1995;Gutierrez et al., 1994) and auditory cortex of aged rats (Caspary et al., 2013;Ling et al., 2005). Research using immunohistochemical techniques to perform cell counts in the hippocampus revealed significant loss of GAD (Stanley & Shetty, 2004) and GABAergic interneurons of aged rats (Stanley et al., 2012). More work utilizing immunostaining to assess age-related changes in levels of GABAergic neurons in the primary visual cortex of young (1–3 years) and old (12 years) cats has also shown that the older cats exhibited a significantly reduced proportion of GABAergic neurons compared to younger cats (Hua et al., 2008). A study of non-human primates similarly observed a significant decrease in GABA levels in the old (21 years) compared to young (4 years) group and an association between age and GABA levels in the posterior cingulate cortex (He et al., 2016). Together, these animal studies provide support for age-related effects throughout the GABAergic system.

GABA can be measured in humans*in vivo*using magnetic resonance spectroscopy (MRS). MRS takes advantage of unique molecular resonances and spectral patterns to classify and measure the quantities of various molecules (De Graaf, 2019;Ende, 2015). Most studies have used a GABA-edited MEGA-PRESS sequence to measure GABA, and due to limitations of the method, the GABA signal is inextricably contaminated with signals from macromolecules. Thus, when referring to GABA as measured using MEGA-PRESS, we will use the term GABA+ to represent GABA plus macromolecules.

Studies using MRS to investigate whether GABA+ declines with age have found mixed results. Some studies have reported lower GABA+ levels in older versus younger adults within frontal, parietal, temporal, and occipital regions (Cassady et al., 2019;Gao et al., 2013;Hermans, Levin, et al., 2018;Lalwani et al., 2019;Simmonite et al., 2019). Other studies have found no age differences in frontal regions (Bai et al., 2015;Porges et al., 2017) or in the cingulate cortex (Aufhaus et al., 2013). And one study even reported an age-related*increase*in a frontal VOI (Ghisleni et al., 2015).

There are at least two issues that could contribute to the discrepancies between studies. The first issue is that all previous human GABA+ MRS studies utilized a cross-sectional design. Thus, any observed differences in GABA+ between age groups could potentially be due to sampling or cohort effects rather than age effects*per se*(Hofer & Sliwinski, 2001;Hofer et al., 2002;Porges et al., 2021). Ideally, a study designed to assess age-related changes in any variable should assess*within*-person change over time (Porges et al., 2021). In the present study, we seek to address this issue by measuring within-person change in GABA levels in multiple brain regions longitudinally.

A second issue with human MRS studies of age differences in GABA+ is the necessity to correct for age-related differences in tissue composition. Research has consistently shown that brain volume declines with age (Hedman et al., 2012;Pini et al., 2016) and that the rates of atrophy differ between tissue type (i.e., gray or white matter) and vary regionally, with frontal and temporal atrophy occurring more rapidly compared to parietal and occipital regions (Fjell et al., 2009;Raz et al., 2005;Walhovd et al., 2005). Moreover, the distribution of GABA across different tissue types is not uniform, with roughly twice as much GABA observed in gray compared to white matter (Harris et al., 2015;Maes et al., 2018). Observed cross-sectional age differences in GABA+ could, therefore, be due to differences in tissue composition rather than the differences in GABA+ itself. For instance,Maes et al. (2018)reported that older participants exhibited lower GABA+ levels compared to younger participants before tissue correction. However, when GABA+ measurements were corrected for tissue composition, the age differences were no longer observed.

Furthermore, the*type*of tissue correction can impact the interpretation of MRS results (Porges et al., 2017). In an experiment designed to assess the impact of different correction types,Porges et al. (2017)observed an age-related decline in GABA+ in a frontal voxel both when no tissue correction was applied and when a simple tissue composition correction for cerebrospinal fluid (CSF) was applied. The CSF-correction (sometimes referred to simply as tissue correction) normalizes the voxel of interest (VOI) by the non-CSF voxel fraction (1-f_CSF_) and therefore assumes that any signal from the CSF portion of the voxel does not contribute to the signal of interest. A more advanced tissue correction approach proposed byHarris et al. (2015), building on an approach byGasparovic et al. (2006), accounts for the different relaxation constants and water visibility between tissue types as well as the ratio of GABA expected in gray to white matter (2:1). To make GABA+ measurements comparable between groups of interest, this correction method goes further to normalize GABA+ measurements by a standard voxel composition created from each group of interest. This correction is often referred to as group α-correction. WhenPorges et al. (2017)applied group α-correction to their previously mentioned results, the relationship between age and frontal GABA+ levels disappeared, suggesting that the initially observed differences were due to differences in tissue composition between the participants. Thus, it is not solely whether tissue correction is applied, but also the*type*of tissue correction applied that can affect the interpretation of MRS measurements of GABA+ in aging humans.

The current study aims to address these two critical issues. Specifically, we conducted a longitudinal study of healthy older adults to account for cohort effects and to have each participant essentially serve as their own control over time. Additionally, we utilized the two most commonly applied types of tissue correction (CSF tissue-correction and group α-tissue correction) to assess the impact on the interpretation of the MRS data. Because most aging studies of GABA observed lower GABA in the older compared to younger groups, we hypothesize that as age increases, GABA+ levels will decrease in all VOIs over time, even after controlling for tissue composition.

## 2 Methods

### 2.1 Participants

Thirty healthy, older adults (*n = *30, 17 females, mean age at initial session = 70.6 ± 4.03 years, range = 65–81 years; mean age at second session = 74.9 ± 4.23 years, range = 68–86 years, average time between sessions = 4.33 years)Table 1were recruited from Ann Arbor, Michigan, and the surrounding area as a part of a large longitudinal study. All participants were proficient English speakers and right-handed, were free from cognitive impairment based on evaluation by a clinical psychiatrist at the Michigan Alzheimer’s Disease Research Center based on the National Alzheimer’s Coordinating Center (NACC) neuropsychological battery (Weintraub et al., 2009). Additionally, participants were screened for MR contraindications and were excluded if pregnant or for any history of neurological or psychiatric disorders, or drug or alcohol abuse. Full exclusion and inclusion criteria can be found inGagnon et al. (2019). All procedures were approved by the University of Michigan Institutional Review Board, and all participants provided written, informed consent.

**Table 1.** Participant demographics.

	Session 1	Session 2
Age	70.6 (4.03)	74.9 (4.23)
Gender	17 Females, 13 Males	
Education, years (SD)	17 (2.32)	

### 2.2 MRS data acquisition

**Fig. 1.**
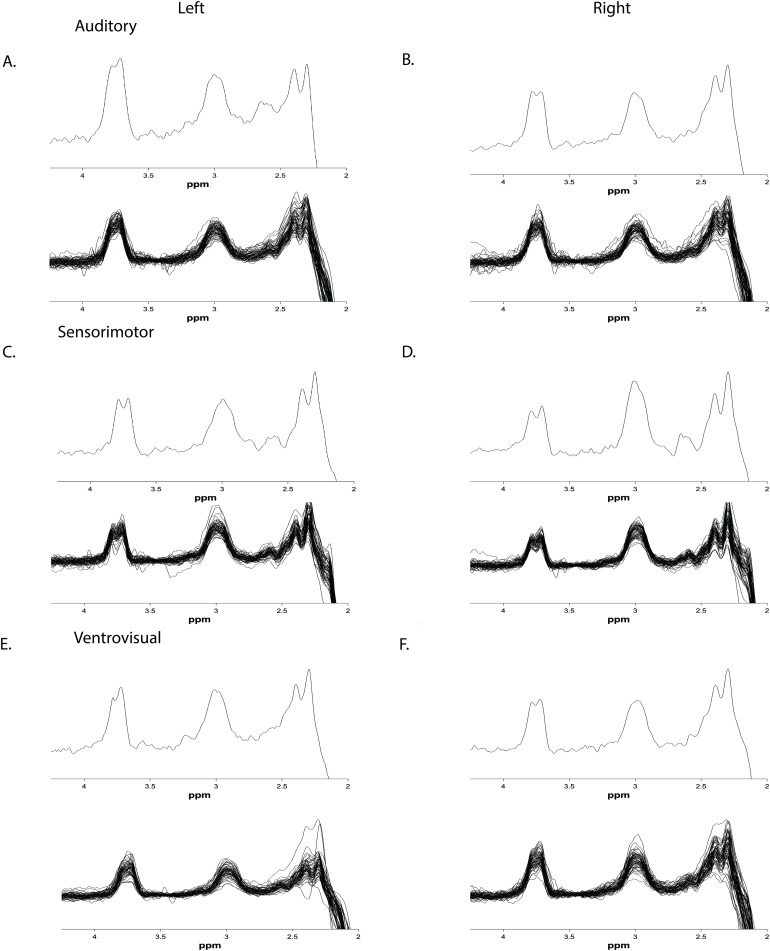
Representative spectrum (top) and all participant spectra (bottom) for left (A) and right (B) auditory VOI, left (C) and right (D) sensorimotor VOIs, and left (E) and right (F) ventrovisual VOIs.

GABA-edited MR spectra were collected from a 3T GE Discovery MR750 with an 8-channel head coil located at the University of Michigan Functional Magnetic Resonance Imaging Laboratory using a MEGA-PRESS sequence (Mescher et al., 1998;Mullins et al., 2014) with the following parameters: TR = 1800 ms; TE = 68 ms (TE1 = 15 ms, TE2 = 53 ms); 256 transients (128 ON interleaved with 128 OFF) of 4096 data points; spectral width = 5 kHz; frequency selective editing pulses (14 ms) applied at 1.9 ppm (ON) and 7.46 ppm (OFF); FOV = 240 × 240 mm; and voxel size = 30 × 30 × 30 mm. Acquisition time for each voxels of interest (VOI) was ~8.5 min. MRS data were collected from voxels placed in the left and right auditory cortex (Fig. 1A), left and right sensorimotor cortex (Fig. 1B), and left and right ventrovisual cortex (Fig. 1C). One participant requested to leave the scanner before the left ventrovisual VOI could be collected for their second timepoint, leaving this VOI with only 29 participants; all 30 participants underwent MRS scanning for the other five VOIs. Additionally, a high-resolution T1-weighted spoiled 3D gradient-echo acquisition (SPGR) image was collected for MRS voxel placement and segmentation with the following parameters: Inversion Time = 500 ms, flip angle = 15°, and field of view = 256 x 256 mm. Segmentation and co-registration of the T1-weighted anatomical image was performed using the SPM12 segmentation function that is integrated into Gannet 3.1 (Ashburner & Friston, 2005).

Data collected from the six cortical VOIs were placed in order to maximize overlap with fMRI activation from visual, auditory, and motor tasks assessed individually for each participant as a part of a larger study (Gagnon et al., 2019). To determine voxel placements, a general linear model (GLM) was performed on fMRI tasks that were conducted as part of the larger Michigan Neural Distinctiveness study (Gagnon et al., 2019), contrasting each condition against rest. Specifically, in a visual task, we computed contrast maps for houses versus fixation and for faces versus fixation. Using these two contrast maps and the T1 structural image, we placed the ventral visual voxels to capture the areas of the average highest activation (highest beta value) for the house and face areas for each hemisphere. For the auditory voxels we used the contrast maps for speech versus no sound and music versus no sound. For the sensorimotor voxels, we placed the left hemisphere voxel to capture activations from the right-hand motor task and the right hemisphere voxel to capture activations from the left-hand motor task. Bilateral VOI placements are shown inFigures 2–3. A dice coefficient analysis confirmed significant overlap between VOI placements between timepoints (seeSupplementary Materialfor full details).

**Fig. 2.**
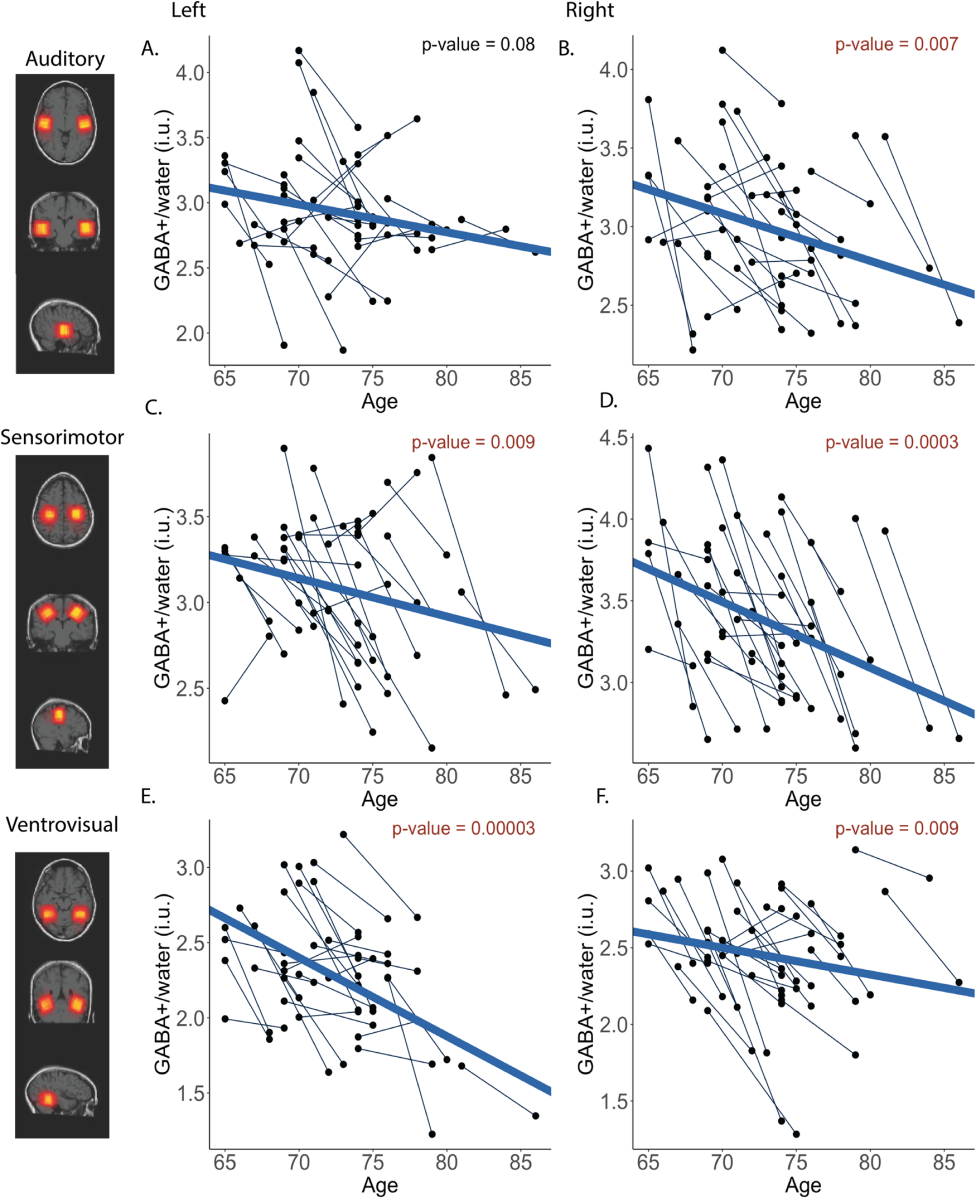
CSF-corrected GABA+ trajectories for left (A) and right (B) auditory VOI, left (C) and right (D) sensorimotor VOIs, and left (E) and right (F) ventrovisual VOIs. Fixed effects of age*p*-values are presented in red if significant and black if not significant.

**Fig. 3.**
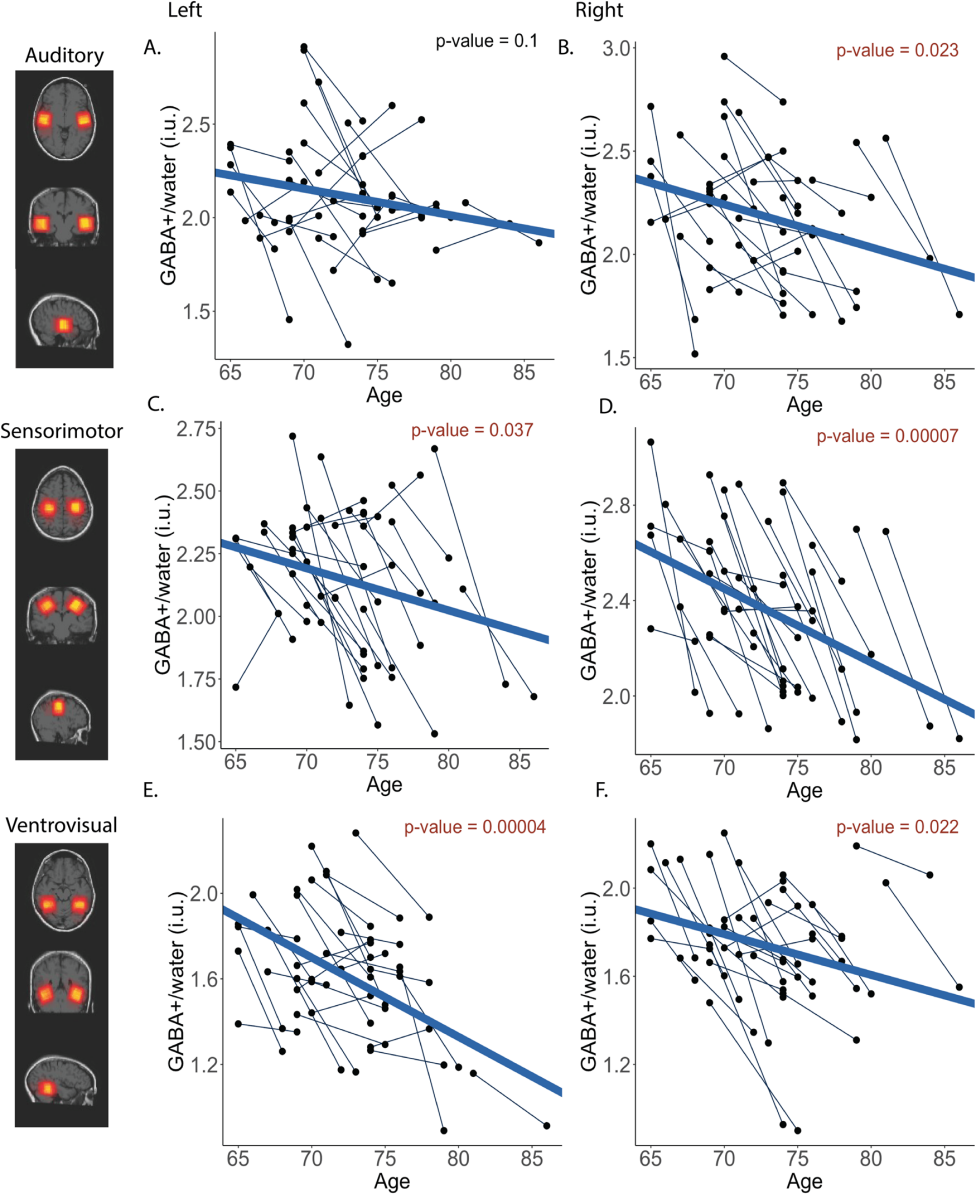
α-TC GABA+ trajectories for left (A) and right (B) auditory VOI, left (C) and right (D) sensorimotor VOIs, and left (E) and right (F) ventrovisual VOIs. Fixed effects of age*p*-values are presented in red if significant and black if not significant.

### 2.3 Quantification of GABA

To estimate GABA+ levels, we utilized the Gannet 3.3.1 MATLAB toolbox (Edden et al., 2014). In this pipeline, time domain data were corrected for phase and frequency using spectral registration (Near et al., 2015), filtered with 3 Hz exponential line broadening, and zero-filled by a factor of 16. A Gaussian-Lorentzian model was then fit to the 3-ppm peak in the difference spectrum, and GABA levels were quantified relative to water in institutional units. Due to the GABA-editing process, significant excitations of extraneous macromolecules have been reported to contribute to the edited GABA signal at 3 ppm (Behar et al., 1994). Thus, as previously mentioned, when referring to GABA, we will use GABA+ to represent GABA plus macromolecules.

### 2.4 MRS data quality

As a measure of quality of fit, Gannet 3.1 calculates a measure of model fit error that equals the ratio of the standard deviation of the fit residual normalized by the amplitude of the fitted peak. Because GABA was referenced to water, Gannet documentation (https://markmikkelsen.github.io/Gannet-docs/data-quality-metrics.html#Fit_error) recommends adding the fit errors for both GABA and water in quadrature. Those values, along with linewidth and signal-to-noise (SNR) ratios are presented in theTable 2below. Generally, fit error of >20% is used as a cutoff for participants that should be excluded from a study (Peek et al., 2020). Average fit error for any VOI did not exceed 4.40% with standard deviations not exceeding 1.87%, with maximum values of fit error for LAUD (8.53%), RAUD (8.94%), LSM (12.31%), RSM (5.13%), LVV (9.70%), and RVV (6.00%) VOIs not exceeding a fit error of 12.31%. To estimate the degree of scanner-related frequency drift and participant motion, the average frequency offset ($\bar{\Delta \delta_{0}}$) was calculated as the mean difference between the observed frequency of the residual water signal in the pre-frequency-corrected sub spectra and the nominal water frequency at 4.68 ppm. Values of$\bar{\Delta \delta_{0}}$for each VOI for each timepoint are shown inTable 2. A representative spectrum and all participant spectra from every VOI is presented inFigure 1.

**Table 2.** Data quality metrics for GABA estimates.

	Timepoint 1				Timepoint 2			
VOI	Fit error (%)	Linewidth (FWHM, Hz)	SNR	Δδ _0_	Fit error (%)	Linewidth (FWHM, Hz)	SNR	Δδ _0_
LAUD	4.40 (1.39)	12.25 (1.48)	16.19 (2.83)	-0.010 (0.01)	4.20 (1.01)	13.61 (2.92)	11.83 (2.70)	-0.018 (0.01)
RAUD	3.83 (1.41)	12.56 (2.36)	15.45 (3.37)	-0.006 (0.02)	4.27 (1.08)	13.47 (3.30)	12.05 (2.60)	0.008 (0.02)
LSM	4.11 (1.87)	10.26 (1.71)	16.83 (2.39)	-0.007 (0.02)	4.15 (0.66)	9.85 (1.29)	13.67 (2.63)	0.003 (0.02)
RSM	3.52 (0.67)	10.35 (1.33)	19.07 (3.42)	-0.010 (0.01)	3.83 (0.71)	9.71 (0.87)	13.81 (2.35)	0.001 (0.02)
LVV	3.92 (1.42)	12.38 (1.97)	14.40 (2.81)	0.002 (0.02)	4.29 (1.28)	12.55 (2.49)	11.58 (3.27)	0.005 (0.02)
RVV	3.47 (0.61)	12.42 (2.63)	16.57 (2.51)	-0.012 (0.02)	4.17 (0.90)	12.55 (2.39)	12.58 (3.38)	0.009 (0.01)

*Note*. Values are presented as means (standard deviations). Full width at half max (FWHM) of water; Signal-to-noise ratio (SNR)); Δδ_0_= average frequency offset.

### 2.5 GABA corrections

The main aims of this study were to measure GABA over time in healthy older individuals, and to assess the effect of common GABA MRS tissue corrections on the interpretation of the results. The primary goal of correction is to remove the effect that tissue composition has on the measurements of GABA+. Therefore, along with uncorrected GABA+ results, the following tissue corrections were performed: (1) CSF tissue correction, accounting for the differential MR tissue relaxation, signal constants, and for the fraction of the voxel that is CSF, and (2) group α-tissue correction, that builds on CSF-correction to account for the different concentrations of GABA+ in gray and white matter, and for the different tissue concentrations across participants (Gasparovic et al., 2006;Harris et al., 2015).

### 2.6 Statistical analyses

Longitudinal changes in GABA+ measurements for all VOIs were analyzed using linear mixed-effects models with random intercepts. Age was included in the model as a fixed effect to assess GABA+ measurement changes with age. Age was centered at 65 due to it being the minimum age of recruitment for this study examining healthy older adults. Changes in gray matter, white matter, and CSF were accounted for using the correction methods mentioned previously. All analyses were conducted in SPSS version 28 (IBM, 2021).

We also constructed another statistical model that incorporates changes in GABA in all the VOIs within a single model and that also accounts for data quality. Specifically, longitudinal changes in GABA+ measurements were analyzed using a linear mixed-effects model with within-subject age change, between-subject age, hemisphere (left or right), and data quality (fit error) included as fixed effects. The model also included random intercept and slope terms to account for the fact that different participants had different initial GABA levels and rates of decline. We also included two covariates to account for the three different regions (visual, auditory, and motor) and these were effect coded, using one of the regions as the reference. For example, when using the auditory region as reference, one covariate would have the value 1 for data in the visual regions, 0 for data in the sensorimotor regions, and -1 for data in the auditory regions, while the other covariate would have the value 1 for the sensorimotor regions, 0 for the visual regions, and -1 for the auditory regions. We ran models using each region as the reference region so that we could get parameter estimates and*p*-values for all three regions.

Lastly, we did not have*a priori*hypotheses about the metabolites that could be analyzed from the OFF experiment. However, as an exploratory analysis, we analyzed the OFF spectra data using Gannet and mixed linear models to investigate longitudinal changes of creatine (Cr), choline (Cho), and*N*-acetylaspartate (NAA) in the left auditory (LAUD), right auditory (RAUD), left sensorimotor (LSM), right sensorimotor (RSM), left ventrovisual (LVV), and right ventrovisual (RVV) voxels of interest (VOIs) in which data were acquired. All exploratory analyses are included in theSupplemental Material.

## 3 Results

Multilevel model results are shown inTables 3–4for CSF-corrected GABA+ and group α-corrected GABA+, respectively. Data for all VOIs and corrections are illustrated inFigures 2–3. For the left auditory VOI (Figs. 2A&3A), age was not a significant predictor of CSF-corrected GABA+ (*b*= -0.02,*p*= .08) or α-corrected GABA+ (*b*= -0.01,*p*= .10). For the right auditory VOI (Figs. 2B&3B), age was a significant predictor of CSF-corrected GABA+ (*b*= -0.03,*p*= .007) and α-corrected GABA+ (*b*= -0.02,*p*= .02).

**Table 3.** Multilevel model results for changes in CSF-corrected GABA+ as a function of age.

CSF-corrected GABA	Effect or model fit	Unstandardized estimate (SE) or fit statistic
Left Auditory VOI	*Fixed Effects:*	
	Intercept	3.10 (.11) ***
	Age	-0.02 (.01)
	*Random Effect:*	
	GABA Intercept	0.05 (.04)
	*Model Fit:*	
	AIC	75.45
Right Auditory VOI	*Fixed Effects:*	
	Intercept	3.30 (.11) ***
	Age	-0.03 (.01) **
	*Random Effect:*	
	GABA Intercept	0.06 (.04)
	*Model Fit:*	
	AIC	75.30
Left Sensorimotor VOI	*Fixed Effects:*	
	Intercept	3.33 (.11) ***
	Age	-0.03 (.01) **
	*Random Effect:*	
	GABA Intercept	0.04 (.03)
	*Model Fit:*	
	AIC	69.86
Right Sensorimotor VOI	*Fixed Effects:*	
	Intercept	3.80 (.13) ***
	Age	-0.05 (.01) ***
	*Random Effect:*	
	GABA Intercept	0.05 (.04)
	*Model Fit:*	
	AIC	84.18
Left Ventrovisual VOI	*Fixed Effects:*	
	Intercept	2.66 (.10) ***
	Age	-0.05 (.01) ***
	*Random Effect:*	
	GABA Intercept	0.07 (.03) *
	*Model Fit:*	
	AIC	52.05
Right Ventrovisual VOI	*Fixed Effects:*	
	Intercept	2.69 (.11) ***
	Age	-0.03 (.01) **
	*Random Effect:*	
	GABA Intercept	0.06 (.03)
	*Model Fit:*	
	AIC	61.31

Note.*p < .05, **p < .01, ***p < .001.

**Table 4.** Multilevel model results for changes in α-TC GABA+ as a function of age.

α-TC GABA	Effect or model fit	Unstandardized estimate (SE) or fit statistic
Left Auditory VOI	*Fixed Effects:*	
	Intercept	2.23 (.10) ***
	Age	-0.01 (.01)
	*Random Effect:*	
	GABA Intercept	0.02 (.02)
	*Model Fit:*	
	AIC	30.29
Right Auditory VOI	*Fixed Effects:*	
	Intercept	2.35 (.08) ***
	Age	-0.02 (.01) *
	*Random Effect:*	
	GABA Intercept	0.01 (.02)
	*Model Fit:*	
	AIC	35.47
Left Sensorimotor VOI	*Fixed Effects:*	
	Intercept	2.28 (.07) ***
	Age	-0.02 (.01) *
	*Random Effect:*	
	GABA Intercept	0.0000008 (.02)
	*Model Fit:*	
	AIC	22.67
Right Sensorimotor VOI	*Fixed Effects:*	
	Intercept	2.67 (.10) ***
	Age	-0.04 (.01) ***
	*Random Effect:*	
	GABA Intercept	0.02 (.02)
	*Model Fit:*	
	AIC	34.62
Left Ventrovisual VOI	*Fixed Effects:*	
	Intercept	1.88 (.08) ***
	Age	-0.04 (.01) ***
	*Random Effect:*	
	GABA Intercept	0.04 (.02) *
	*Model Fit:*	
	AIC	15.10
Right Ventrovisual VOI	*Fixed Effects:*	
	Intercept	1.88 (.07) ***
	Age	-0.02 (.01) *
	*Random Effect:*	
	GABA Intercept	0.01 (.02)
	*Model Fit:*	
	AIC	19.76

Note.*p < .05, ***p < .001.

Age was also a significant predictor of GABA+ in the left sensorimotor cortex for both corrections (Figs. 2C&3C; CSF-corrected GABA+ [*b*= -0.03,*p*= .009], and α-corrected GABA+ [*b*= -0.02,*p*= .04]) and this was also observed in the right sensorimotor VOI (Figs. 2D&3D; CSF-corrected GABA+ [*b*= -0.05,*p*= .0003], and α-corrected GABA+ [*b*= -0.04,*p*= .00007]).

Finally, similar longitudinal declines in GABA+ were observed in the left ventrovisual VOI (Figs. 2E&3E; CSF-corrected GABA+ [*b*= -0.05,*p*= .00003], and α-corrected GABA+ [*b*= -0.04,*p*= .00004]). For the right ventrovisual VOI (Figs. 2F&3F), age was a significant predictor of CSF-corrected GABA+ (*b*= -0.03,*p*= .009) and α-corrected GABA+ (*b*= -0.02,*p*= .022).

The random intercepts variance was significant in a few VOIs (CSF-corrected GABA+ in the left ventrovisual VOI [*p*= .02]; α-corrected GABA+ in the left ventrovisual VOI [*p*= .04]), suggesting that participants exhibited different levels of GABA+ at the age of 65 in these cases.

**Fig. 4.**
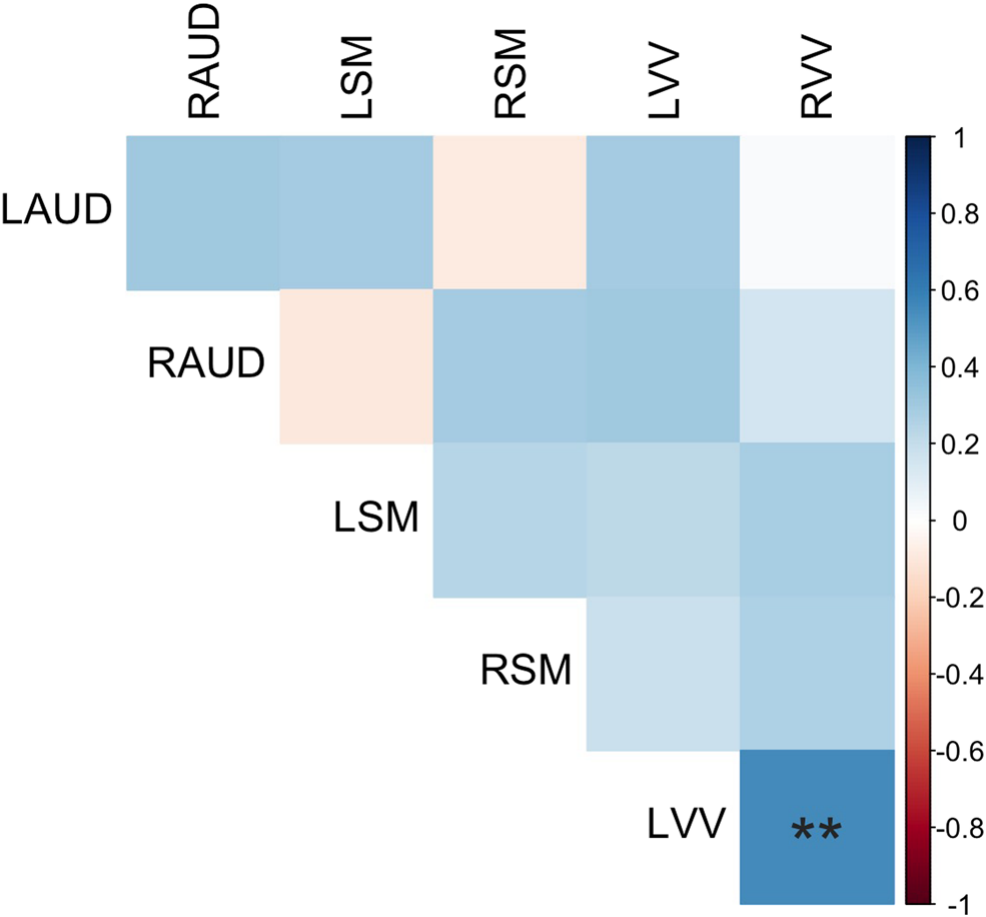
Correlation matrix showing relationships between GABA slopes across VOIs. Significant relationships are flagged, **p < .01.

To further explore the question of whether certain regions exhibit steeper declines, we investigated whether GABA+ levels declined at a different rate across the different VOIs. To do this, we computed the rate of change in GABA+ (slope) for each participant in each VOI and then assessed for regional differences by computing a one-way ANOVA with GABA+ slopes as the dependent variable and VOI as group factor. The analysis revealed a significant main effect of group (*p*= .004). A post-hoc Tukey test revealed that the right sensorimotor area exhibited significantly steeper declines when compared to the left auditory (mean differences of 0.077,*p*= .002) and left ventrovisual (mean difference of 0.070,*p*= .011) VOIs. No other VOIs significantly differed from each other in GABA+ slope. To investigate whether any regions change together or independently, correlations were performed for each participant’s GABA+ slope across VOIs. Results showed that only the left and right ventrovisual VOIs had slopes that significantly correlated with each other (*r*= .57,*p*= .001;Fig. 4). These analyses suggest that declines in GABA occur regionally, and the declines in the ventrovisual area tend to occur at a similar bilateral pace.

We found similar results when we fit all the data using a single statistical model that incorporates all the VOIs within a single model and that also accounts for data quality. Once again, the effect of longitudinal within-subject age change was significant and negative (*b*= -0.07,*p*< .001). And this was true despite the fact that there was no significant cross-sectional effect of between-subject age (*b*= -0.005,*p*= .045). These results suggest that there is no cross-sectional effect of age across people in our sample, but that there is a longitudinal effect of age*within*people. Additionally, there was an effect of hemisphere, with the left hemisphere VOIs having significantly less GABA than the right hemisphere (*b*= -0.06,*p*< .001). Across regions, the sensorimotor region had higher than average GABA values (*b*= 0.22,*p*< .001), the ventrovisual had lower than average values (*b*= -0.35,*p*< .001), and the auditory regions had higher than average values (*b*= 0.13,*p*< .001). Lastly, there was an effect of data quality on the measurement of GABA, in that the higher the fit error, the lower the estimated level of GABA (*b*= -0.03,*p*= .006).

It is important to note that fit error was not higher than the 20% recommended cut off (Peek et al., 2020) in any participant, with the highest recorded fit error in any participant being 12.31%. Furthermore, the effect of within-subject age change on GABA remains significant after including fit error in the model. We also tested whether fit error interacted with region, but it did not. Together, the results from the full model analysis confirm that GABA levels decline within individuals as they get older, even after controlling for fit error and despite the fact that there was no cross-sectional effect of age on GABA (further demonstrating the importance of distinguishing longitudinal from cross-sectional results, both theoretically and statistically).

To investigate whether there are age-related changes in any of the QA measures, mixed linear model analyses were performed on each of the QA metrics similar to analysis of age-related changes in GABA. Age was not a significant predictor of fit error in the LAUD (*p*= .56), RAUD (*p*= .27), LSM (*p*= .86), RSM (*p*= .11), LVV (*p*= .28), or RVV (*p*= .36) VOIs. Nor was age a significant predictor of linewidth in LAUD (*p*= .25), RAUD (*p*= .24), LSM (*p*= .75), RSM (*p*= .34), LVV (*p*= .64), or RVV (*p*= .15) VOIs. Lastly, age was not a significant predictor of average frequency offset (Δδ_0_) for the LAUD (*p*= .06), RAUD (*p*= .80), LSM (*p*= .65), RSM (*p*= .07), LVV (*p*= .83), or RVV (*p*= .20) VOIs.

## 4 Discussion

The main aim of the current study was to investigate within-person, age-related changes of GABA+ in various brain regions over time and after the application of two commonly utilized methods of tissue correction. Linear mixed-effects model results showed that age was a significant predictor of CSF-corrected GABA+ in all the VOIs except for the left auditory VOIs. Similarly, after group α-tissue correction, age remained a significant predictor of change in GABA+ in all VOIs except for the left auditory cortex. Further, a model consisting of GABA+ measurements from all VOIs found no cross-sectional effect of age across people in our sample, but that there was a longitudinal effect of age*within*people. These results fill a gap in the literature by providing evidence for the hypothesis that GABA+ levels decline longitudinally with age in auditory, sensorimotor, and ventrovisual regions, even after considering age-related tissue changes.

These results are consistent with theories that assume that age-related declines in GABA and/or in cortical inhibition are an important factor in age-related cognitive decline. For example, the inhibition theory of aging postulates that a reduction in the ability of healthy older adults to control and inhibit excessive neural activation for the purpose of guiding thought and action to complete a goal is a major factor in age-related cognitive decline (Hasher, 2015;Hasher & Campbell, 2020;Hasher & Zacks, 1988). Evidence has consistently shown that older adults are much less able to inhibit distracting stimuli for visual and auditory information compared to younger adults (Jost et al., 2011;McNab et al., 2015;Stevens et al., 2008;Zanesco et al., 2020). The reduction in the ability to inhibit distractions has been shown to detrimentally affect other aspects of cognition. For instance,Zanesco et al. (2020)found that older adults were less able to inhibit distractor interference during a working memory task compared to younger adults, although they still performed better than adolescent participants. Similarly,Hermans, Leunissen, et al. (2018)observed that lower levels of GABA in older adults were associated with slower performance on a motor inhibition task. Neuroimaging studies utilizing fMRI provide further evidence that older adults exhibit a decline in their ability to suppress activation for irrelevant stimuli. For instance,Gazzaley et al. (2005)observed that when older and younger adults were shown sequences of faces and scenes, and were told to ignore the scenes, the older adults exhibited higher activation in a scene-selective ROI compared to younger adults. The present results provide biological and longitudinal evidence for an age-related decline in GABA that could contribute to age-related deficits in inhibition.

The present results are also consistent with the theory of age-related neural dedifferentiation. This theory posits that a decrease in the distinctiveness of neural representations (i.e., neural dedifferentiation) contributes to functional and cognitive decline in healthy aging (Koen & Rugg, 2019;D. C. Park et al., 2004). Studies have consistently shown that neural activation patterns elicited by different categories of sensory stimuli are less distinctive and more confusable in older versus younger adults (Carp et al., 2011;Cassady et al., 2020;Chamberlain et al., 2021;Lalwani et al., 2019;D. C. Park et al., 2004) and less distinctive neural representations have been associated with age-related behavioral impairments, both theoretically (Li & Lindenberger, 1999;Li & Sikstrom, 2002;Li et al., 2000,^2001^) and empirically (Carp et al., 2010;J. Park et al., 2010;Sommer et al., 2019).

A reduction in GABA levels is hypothesized to cause neural dedifferentiation by undermining the ability of competing cortical regions to inhibit each other, and thus, creating less distinct neural representations. Support for this hypothesis comes from studies that have observed an association between individual differences in GABA and neural distinctiveness. For example, studies have observed age-related declines in GABA levels in auditory cortex (Lalwani et al., 2019), sensorimotor regions (Cassady et al., 2019), and in the ventrovisual cortex (Chamberlain et al., 2021). Furthermore, in each of these studies, lower levels of GABA in healthy older adults were associated with other neural and behavioral measures. Specifically,Lalwani et al. (2019)observed that lower levels of GABA in the auditory cortex were associated with less distinct brain activation patterns for music versus speech stimuli;Cassady et al. (2019)observed that lower levels of GABA in the sensorimotor regions of healthy older adults were associated with less segregated networks and worse performance on a sensorimotor tapping task; andChamberlain et al. (2021)observed that lower levels of GABA in the ventrovisual cortex of healthy older adults were associated with less distinct patterns of activations for faces and houses. However, hitherto, it was unknown whether GABA levels declined within individuals or if cross-sectional group differences were due to cohort or other extraneous effects. The present study provides longitudinal evidence for an age-related decline in GABA that could contribute to age-related declines in inhibitory function and in neural distinctiveness.

### 4.1 Limitations

First, this study was designed to address the limitations of previous cross-sectional studies that may or may not have applied tissue correction to their measurements of GABA. Nevertheless, it remains subject to the same limitations that are inherent to measuring GABA with MRS. Specifically, the size of the VOIs in the study is relatively large (3 cm^3^) and so they probably include cortical regions unrelated to the tasks designed to elicit fMRI activity in those regions. Additionally, MRS measures of GABA do not distinguish between intra- and extracellular GABA and do not measure GABA activity but only GABA concentration (Stagg et al., 2011). Another limitation inherent to MRS acquisition at 3T using MEGA-PRESS is the concomitant excitation of the coedited macromolecule signal that has been observed to contribute to the GABA signal at 3 ppm (Mullins et al., 2014;Puts & Edden, 2012). Macromolecular (MM) contamination could be suppressed by applying symmetrical editing pulses about the MM signal (Henry et al., 2001); however, it is important to note that in a multi-site study comparing GABA+ and MM-suppressed GABA measurements (Mikkelsen et al., 2017), the MM-suppressed GABA measurements showed increased variability across sites whereas GABA+ measurements exhibited strong agreement across sites, suggesting GABA+ measurements might be more reliable. The sample size was also relatively small and prevented examination of factors (besides age) that could be important for predicting GABA+ decline (e.g., those linked to educational background or sex/gender). Another limitation is that no analyses with behavioral or cognitive assessments were performed in this study. Thus, it is still not clear whether the observed longitudinal declines in GABA are directly associated with age-related changes in behavior. We have collected a number of behavioral measures and plan to investigate them in a future study when our sample size is larger. Lastly, it is possible that an age-related decline in GABA starts earlier in life for some of the participants, which could also explain the significant difference in intercepts observed in this study. Additional timepoints (i.e., more than two) could allow for better characterization of the age-related trajectory of GABA.

## 5 Conclusion

Findings from the current study provide support for the hypothesis that GABA levels decline globally in healthy aging. Furthermore, these results were not due to tissue changes or atrophy that are associated with healthy aging. They were also observed longitudinally within people and were not due to cross-sectional differences between people of different ages. These results have implications for models of inhibition and neural distinctiveness that posit that GABA declines contribute to age-related functional and cognitive decline.

## Supplementary Material

Supplementary Material

## Data Availability

The code used to analyze the MRS data in this study is freely available as a toolbox and can be found here:https://markmikkelsen.github.io/Gannet-docs/index.html. Data are available upon request.
