# Supplementary material for: GABA levels decline with age: A longitudinal study
Source: Imaging Neurosci (Camb). 2024 Jul 15;2:imag-2-00224. doi: 10.1162/imag_a_00224 (PMC12272259; doi:10.1162/imag_a_00224)
Supplement: Supplementary Material [file imag_a_00224-supp.pdf]

Supplemental Material

Supplemental Analyses

Analysis of OFF spectrum metabolites from the edited MEGA-PRESS sequence

We did not have *a priori* hypotheses about the metabolites that could be analyzed from the OFF experiment. However, as an exploratory analysis and per the suggestion of the reviewer, we analyzed the OFF spectra data using Gannet and mixed linear models to investigate longitudinal changes of creatine (Cr), choline (Cho), and *N*-acetylaspartate (NAA) in the left auditory (LAUD), right auditory (RAUD), left sensorimotor (LSM), right sensorimotor (RSM), left ventrovisual (LVV), and right ventrovisual (RVV) voxels of interest (VOIs) in which data were acquired. Because the **α**-tissue correction strategy is specific to GABA and we still wanted to control for changes in tissue composition for the OFF-spectra metabolites, the CSF tissue correction proposed by Gasparovic et al. (2006) was used for all OFF-spectra metabolites, referenced to water.

Results from the mixed linear models showed that age was a significant predictor of CSF-corrected Cr in the RAUD (b = -0.10, p = 0.045), the LSM (b = -0.10, p = 0.003), the RSM (b = -0.15, p = 0.0002), the LVV (b = -0.07, p = 0.026), and in the RVV (b = -0.10, p = 0.004) VOIs but not in the LAUD (b = 0.04, p = 0.343) VOI (Supplemental Figure 1).

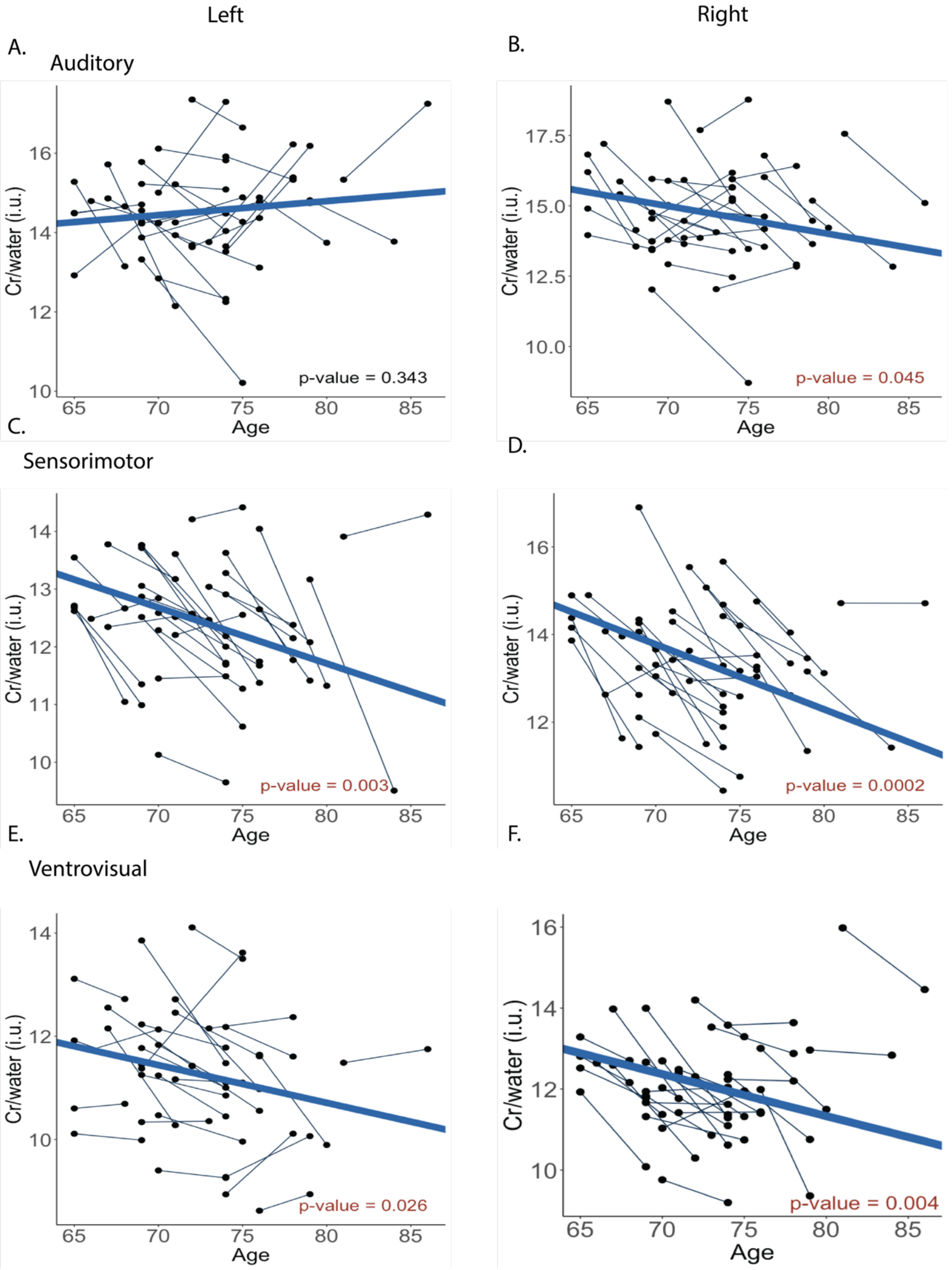

Supplemental Figure 1. CSF-corrected creatine (Cr) trajectories for left (A) and right (B) auditory VOI, left (C) and right (D) sensorimotor VOIs, and left (E) and right (F) ventrovisual VOIs. Significant models for age predicting change in Cr are indicated with *p* values in red and non-significant models in black.

For CSF-corrected Cho, age was a significant predictor in the RAUD (b = -0.03, p = 0.010), the RSM (b = -0.02, p = 0.004), the LVV (b = -0.02, p = 0.001), and in the RVV (b = -0.03, p = 0.002) VOIs but in the LAUD (b = 0.00, p = 0.975) or LSM (b = -0.01, p = 0.058) VOIs (Supplemental Figure 2).

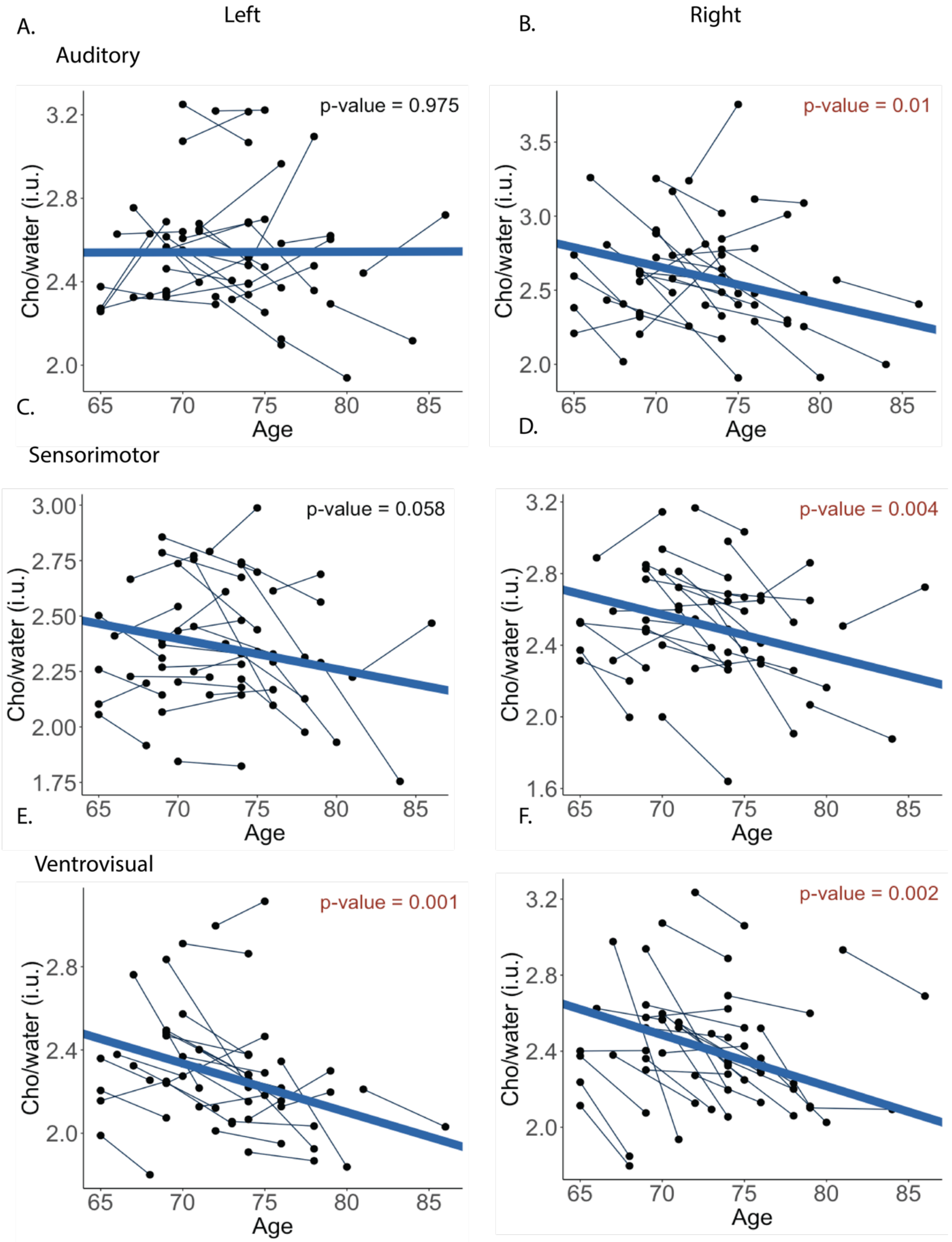

Supplemental Figure 2. CSF-corrected choline (Cho) trajectories for left (A) and right (B) auditory VOI, left (C) and right (D) sensorimotor VOIs, and left (E) and right (F) ventrovisual VOIs. Significant models for age predicting change in Cho are indicated with *p* values in red and non-significant models in black.

For CSF-corrected NAA, age was a significant predictor in only the LSM VOI ($b = -0.06$, $p = 0.047$), and not in the LAUD ($b = -0.07$, $p = 0.159$), RAUD ($b = -0.02$, $p = 0.700$), RSM ($b = -0.06$, $p = 0.132$), LVV ($b = -0.05$, $p = 0.274$), or RVV ($b = -0.07$, $p = 0.109$) VOIs (Supplemental Figure 3).

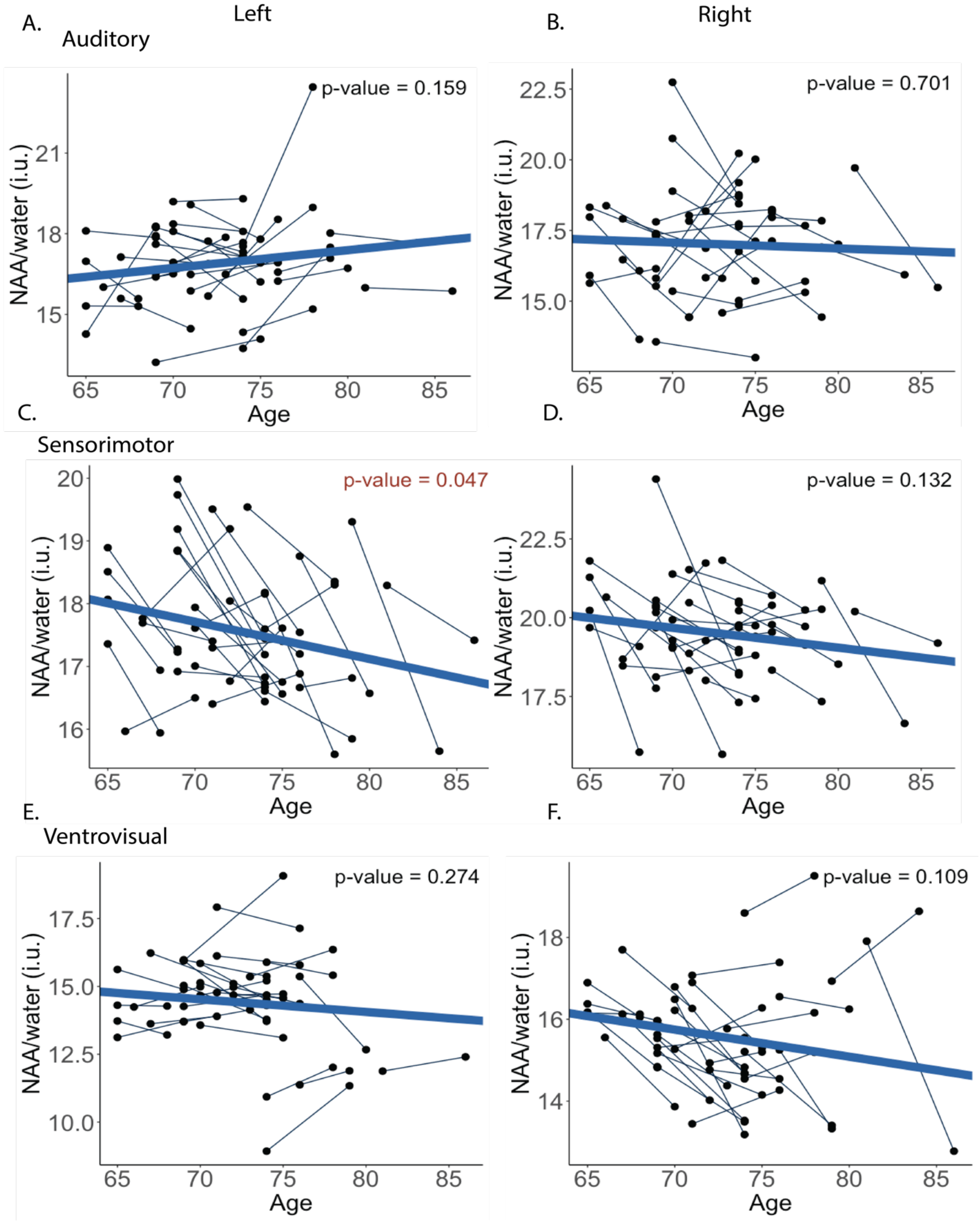

Supplemental Figure 3. CSF-corrected *N*-acetylaspartate (NAA) trajectories for left (A) and right (B) auditory VOI, left (C) and right (D) sensorimotor VOIs, and left (E) and right (F) ventrovisual VOIs. Significant models for age predicting change in NAA are indicated with *p* values in red and non-significant models in black.

Investigating longitudinal changes in tissue fraction

Gray matter (GM) and white matter (WM) fractions for every VOI were computed and were investigated using mixed linear models similar to the main GABA analyses. Results showed that age was a significant predictor of GM in the left auditory VOI ($b = -0.003$, $p = 0.013$) and in the right auditory VOI ($b = -0.003$, $p < 0.001$) but not in any other VOI. Age was a significant predictor of WM in the right ventrovisual VOI ($b = -0.003$, $p = 0.02$) and no other VOI (Supplemental Figures 4 & 5 below).

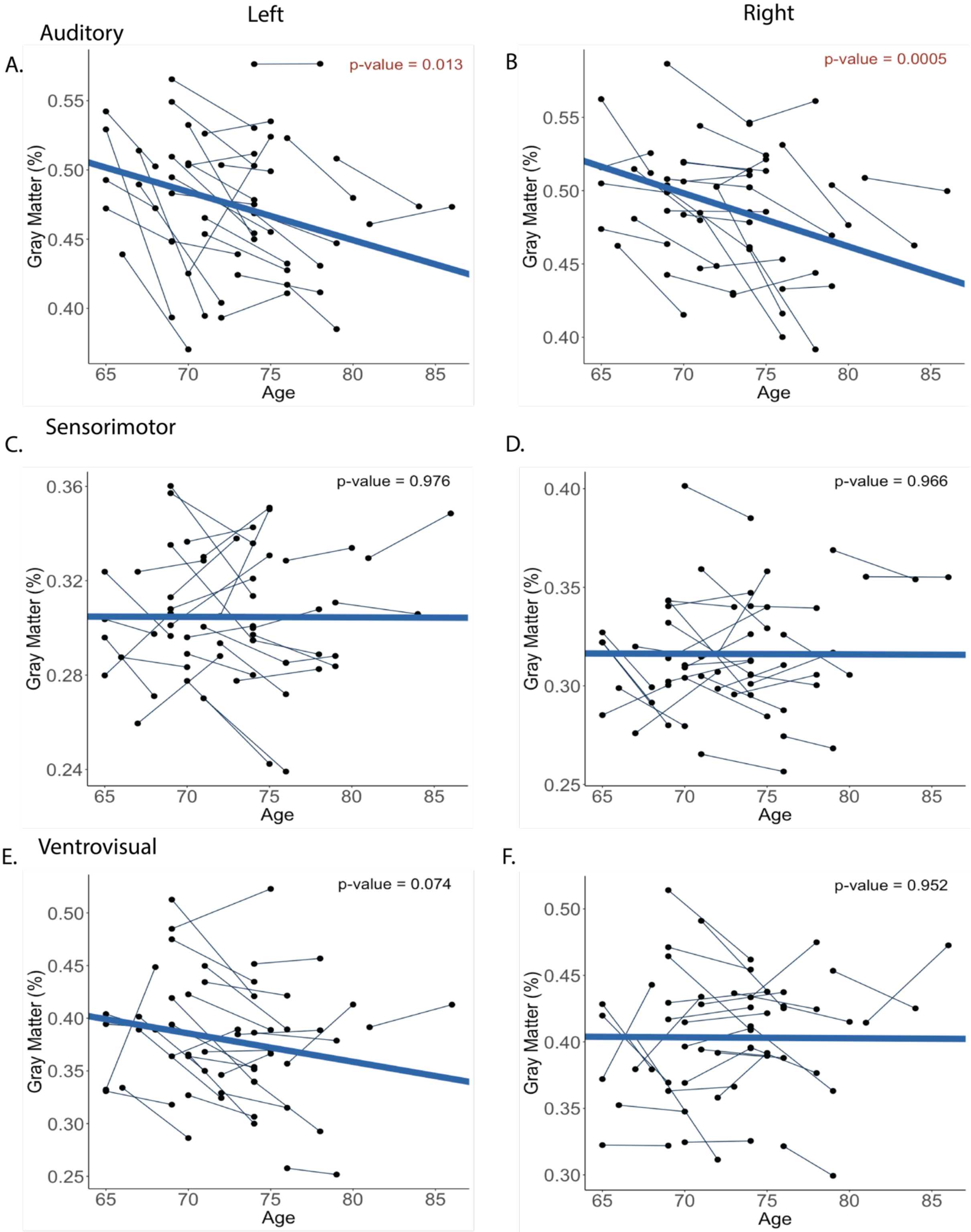

Supplemental Figure 4. Gray matter (GM) fraction trajectories for left (A) and right (B) auditory VOI, left (C) and right (D) sensorimotor VOIs, and left (E) and right (F) ventrovisual VOIs. Significant models for age predicting change in GM are indicated with *p* values in red and non-significant models in black.

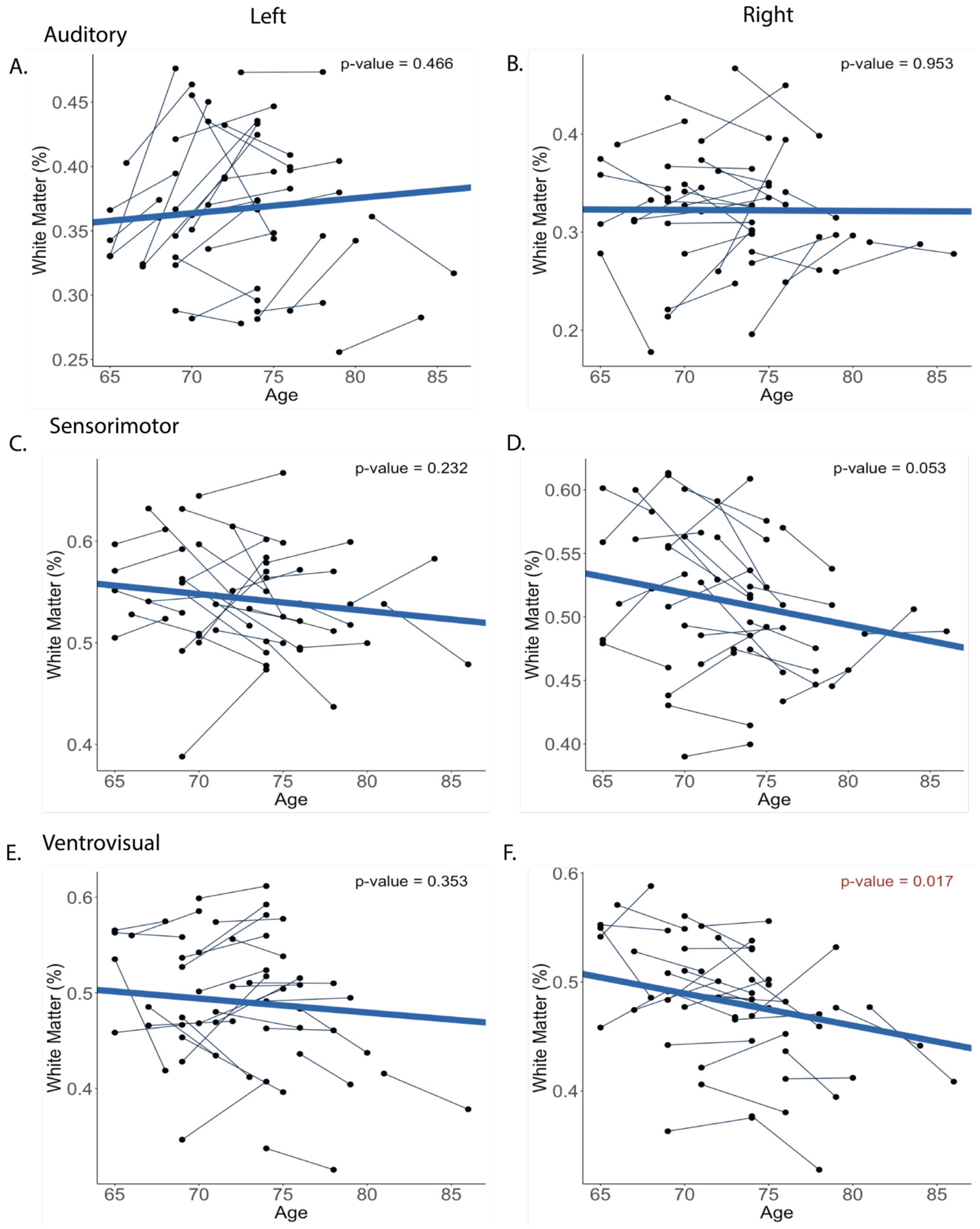

Supplemental Figure 5. White matter (WM) fraction trajectories for left (A) and right (B) auditory VOI, left (C) and right (D) sensorimotor VOIs, and left (E) and right (F) ventrovisual VOIs. Significant models for age predicting change in WM are indicated with *p* values in red and non-significant models in black.

Additionally, the change in GABA over time in relation to the change in GM and WM over time was assessed by computing the slopes for GABA, GM, WM and then using regression models to investigate the

impact that tissue change has on the change in GABA across the different VOIs. Specifically, the change in GABA over time was regressed on to the change in GM or WM over time. Results showed that only change in WM in the right auditory VOI (*p* = .002) and in the right sensorimotor VOI (*p* = .045; Supplemental Figure 6 below) was able to predict the change in GABA whereas the change in GM was not able to predict the change in GABA in any of the VOIs.

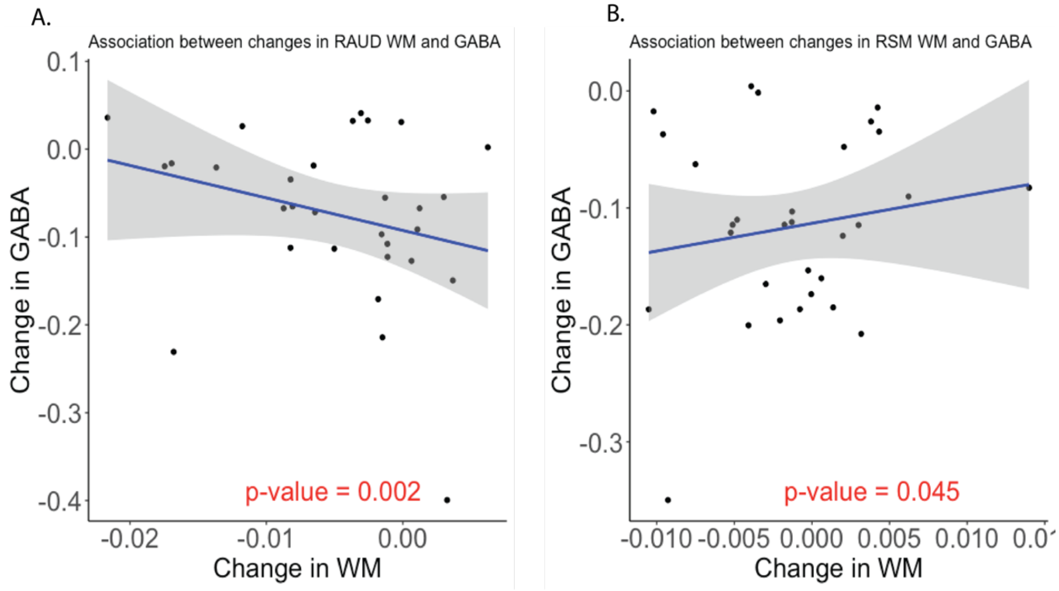

Supplemental Figure 6. Change in GABA predicted by change in white matter (WM) in the right auditory VOI (A) and in the right sensorimotor VOI (B). Standard error of the fixed effect is shown in the shaded area.

Uncorrected-GABA+ Analyses

Uncorrected GABA+ results are presented here (Supplemental Figure 7) so that longitudinal results can still be compared to previous studies of GABA that did not utilize tissue correction strategies. Multilevel model results are shown in Supplemental Table 1 for uncorrected GABA+. Age was a significant predictor of uncorrected GABA+ in the left auditory (b =-0.02, p = 0.01), right auditory (b = -0.02, p = 0.001), left sensorimotor (b = -0.02, p = 0.01), right sensorimotor (b = -0.03, p = 0.00003), left ventrovisual (b = -0.04, p = 0.0000006) and right ventrovisual (b = -0.03, p = 0.0004) VOIs.

The random intercepts variance was significant for uncorrected GABA+ in the left auditory VOI (p = 0.03) and left ventrovisual VOI (p = 0.003), suggesting that participants exhibited different levels of uncorrected GABA+ at the age of 65 in these cases.

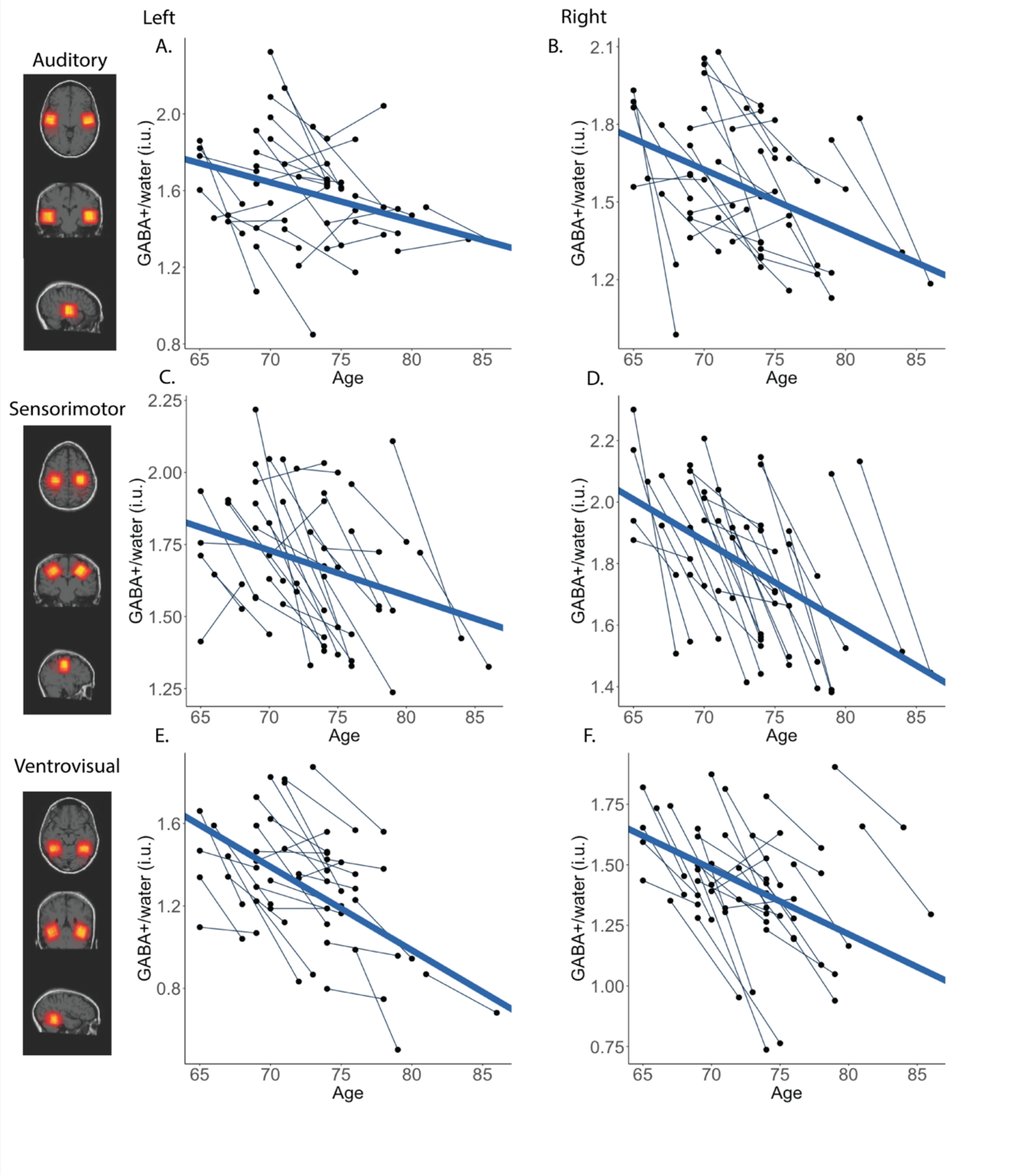

Supplemental Figure 7. Uncorrected GABA+ trajectories for left (A) and right (B) auditory VOIs, left (C) and right (D) sensorimotor VOIs, and left (E) and right (F) ventrovisual VOIs.

Supplemental Table 1. Multilevel model results for changes in uncorrected GABA+ as a function of age

Uncorrected GABA	Effect or Model Fit	Unstandardized Estimate (SE) or Fit Statistic
Left Auditory VOI	*Fixed Effects:*	
	Intercept	1.74 (.07)***
	Age	-0.02 (.01)*
	Random Effect:	
	GABA Intercept	0.03 (.01)*
	Model Fit:	
	AIC	10.87
Right Auditory VOI	*Fixed Effects:*	
	Intercept	1.74 (.07)***
	Age	-0.02 (.01)***
	Random Effect:	
	GABA Intercept	0.01 (.01)
	Model Fit:	
	AIC	6.69
Left Sensorimotor VOI	*Fixed Effects:*	
	Intercept	1.81 (.06)***
	Age	-0.02 (.01)**
	Random Effect:	
	GABA Intercept	0.001 (.01)
	Model Fit:	
	AIC	-1.59
Right Sensorimotor VOI	*Fixed Effects:*	
	Intercept	2.06 (.10)***
	Age	-0.03 (.01)***
	Random Effect:	
	GABA Intercept	0.01 (.01)
	Model Fit:	
	AIC	-2.63
Left Ventrovisual VOI	*Fixed Effects:*	
	Intercept	1.59 (.07)***
	Age	-0.04 (.01)***
	Random Effect:	
	GABA Intercept	0.05 (.02)***
	Model Fit:	
	AIC	-0.94
Right Ventrovisual VOI	*Fixed Effects:*	
	Intercept	1.62 (.07)***
	Age	-0.03 (.01)***
	Random Effect:	
	GABA Intercept	0.03 (.02)
	Model Fit:	
	AIC	4.26

Note. *$p < 0.05$, **$p < 0.01$, ***$p < 0.001$

Glx Analysis

Glx estimates for every VOI were computed and were investigated using mixed linear models similar to the main GABA analyses. Because the **α**-tissue correction strategy is specific to GABA and we still wanted to control for changes in tissue composition, CSF tissue correction proposed by Gasparovic et al. (2006) was used

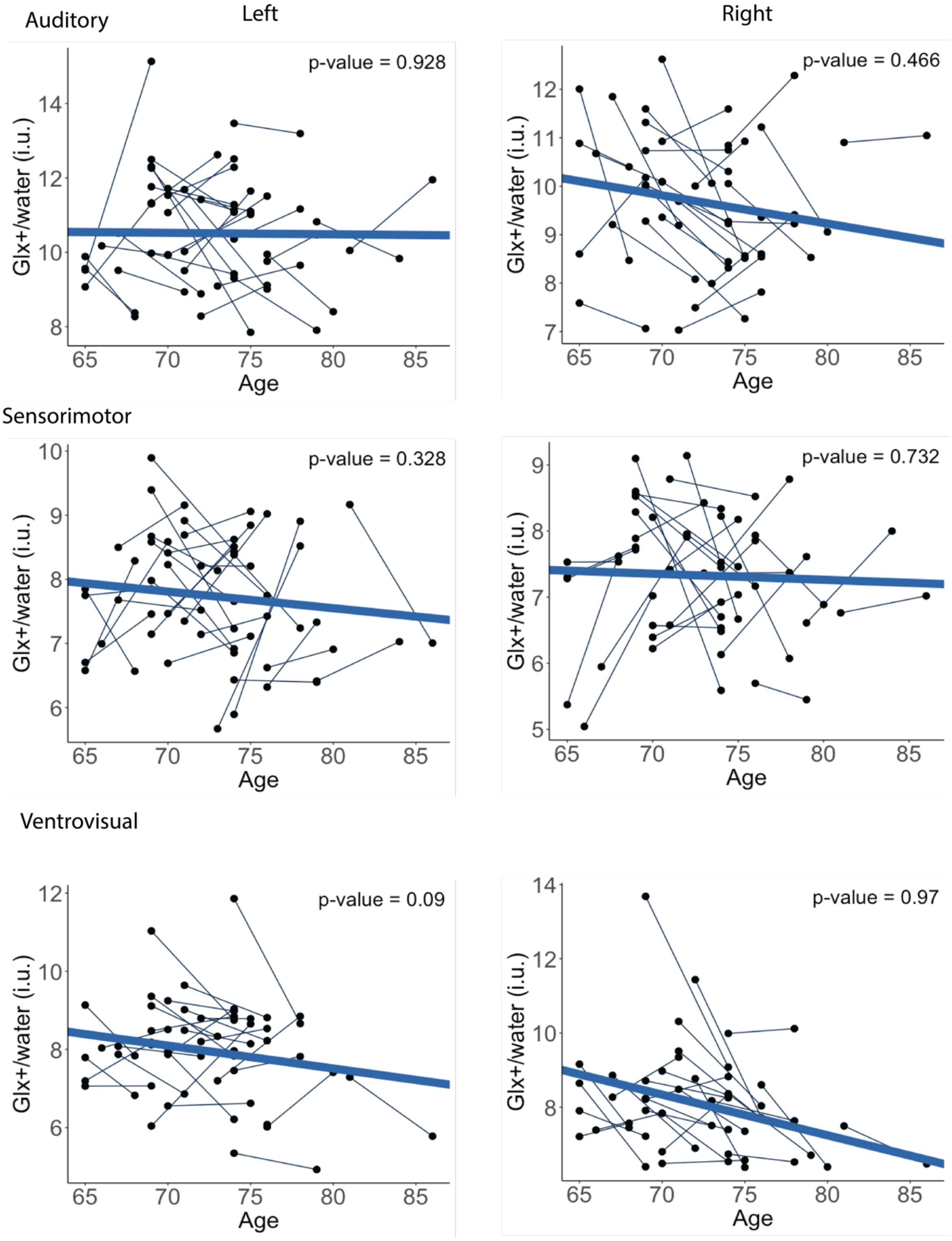

Supplemental Figure 7. Glx trajectories for left (A) and right (B) auditory VOI, left (C) and right (D) sensorimotor VOIs, and left (E) and right (F) ventrovisual VOIs. Significant models for age predicting change in Glx are indicated with *p* values in red and non-significant models in black.

to correct Glx estimates, referenced to water. Results showed that age was not a significant predictor of Glx in any of the VOIs (Supplemental Figure 8 below).

VOI overlap Dice Coefficient Analysis

To assess overlap of VOI placements between longitudinal sessions, we computed Dice overlap coefficients (DOC) between VOI placements at timepoint 1 with placements at timepoint 2 within-subjects. Calculations of DOCs were done according to Bai et al. (2017). Briefly, VOI masks were binarized and transformed into MNI space. Then, quantification of VOI placement overlap within-subjects was performed using the DOC defined as the intersection volume, divided by the mean volume of the two voxels: the DOC ranges from 0 to 1, with 1 being complete overlap. DOC can therefore by multiplied by 100 to represent percentage overlap. The overlap between the 1st and 2nd timepoint VOIs were 65% ± 8.3% for the left auditory VOI, 66% ± 6.7% for the right auditory VOI, 63% ± 6.5% for the left sensorimotor VOI, 59% ± 11.2% for the right sensorimotor VOI, 61% ± 9.5% for the left ventrovisual VOI, and 61% ± 10.2% for the right ventrovisual VOI. At first, these overlap results may seem low but a 65% overlap is equivalent to a 10% (or 3 mm of one direction of a 3 cm^3 voxel) displacement. Considering voxels are placed using T1-weigthed images with 1 mm^3 resolution, the upper limit of precision would be 1 mm in each direction. Furthermore, in separate study designed to assess VOI placement replication within-subject the authors found only an 85% DOC between VOIs placed within the same session. Additionally, a limitation of using the DOC in standard space is that even two VOIs with identical position and orientation would not provide 100% overlap unless brain volumes are also identical.
